# Development of an Improved Carotenoid Extraction Method to Characterize the Carotenoid Composition under Oxidative Stress and Cold Temperature in the Rock Inhabiting Fungus *Knufia petricola* A95

**DOI:** 10.3390/jof4040124

**Published:** 2018-11-09

**Authors:** Kerstin Flieger, Nicole Knabe, Jörg Toepel

**Affiliations:** 1 Department of Plant Physiology, Institute of Biology, Leipzig University, Johannisallee 21-23, 04103 Leipzig, Germany; k.flieger@uni-leipzig.de; 2 Department of Materials & Environment, Bundesanstalt für Material-forschung und-prüfung, BAM, Unter den Eichen 87, 12205 Berlin, Germany; knanic@hotmail.com; 3 Department of Solar Materials, Applied Biocatalytics, Helmholtz Centre for Environmental Research, Permoser Strasse 15, 04318 Leipzig, Germany

**Keywords:** *Knufia petricola* A95, HPLC analysis, carotenoids, black yeast, didehydrolycopene

## Abstract

Black yeasts are a highly specified group of fungi, which are characterized by a high resistance against stress factors. There are several factors enabling the cells to survive harsh environmental conditions. One aspect is the pigmentation, the melanin black yeasts often display a highly diverse carotenoid spectrum. Determination and characterization of carotenoids depend on an efficient extraction and separation, especially for black yeast, which is characterized by thick cell walls. Therefore, specific protocols are needed to ensure reliable analyses regarding stress responses in these fungi. Here we present both. First, we present a method to extract and analyze carotenoids and secondly we present the unusual carotenoid composition of the black yeast *Knufia petricola* A95. Mechanical treatment combined with an acetonitrile extraction gave us very good extraction rates with a high reproducibility. The presented extraction and elution protocol separates the main carotenoids (7) in *K. petricola* A95 and can be extended for the detection of additional carotenoids in other species. *K. petricola* A95 displays an unusual carotenoid composition, with mainly didehydrolycopene, torulene, and lycopene. The pigment composition varied in dependency to oxidative stress but remained relatively constant if the cells were cultivated under low temperature. Future experiments have to be carried out to determine if didehydrolycopene functions as a protective agent itself or if it serves as a precursor for antioxidative pigments like torulene and torularhodin, which could be produced after induction under stress conditions. Black yeasts are a promising source for carotenoid production and other substances. To unravel the potential of these fungi, new methods and studies are needed. The established protocol allows the determination of carotenoid composition in black yeasts.

## 1. Introduction

A wide variety of natural pigments are produced by a wide spectrum of organisms, including bacteria, plants, and fungi. Carotenoids are the main pigments besides the essential photosynthetic chlorophyll pigments. The carotenoid synthesis pathways of most organisms share two steps, which start with the synthesis of geranylgeranyl pyrophosphate (GGPP) from the head-to-tail condensation of four C_5_ isoprene units and the tail-to-tail condensation of two GGPP units to produce the colorless precursor phytoene. These reactions are catalyzed by the enzymes GGPP synthase and phytoene synthase respectively. The introduction of conjugated double bonds into the phytoene backbone yields molecules able to absorb visible light and provides the characteristic yellow, orange to red colors of the carotenoids. Four desaturation steps are necessary for the conversion of phytoene to the maximally de-saturated red lycopene. These steps are carried out by a single enzyme [1,2], a carotene cyclase encoded by Al-2 gene in *Neurospora crassa*. Similar genes were found also in *Exophiala* strains [3]. Later reactions including cyclisation, isomerization, hydroxylation, and oxidation result in the more than 600 different natural carotenoids known so far [1,4,5]. Those can be divided into a class of hydrocarbons (carotenes) and their oxygenated derivatives (xanthophylls). In general, carotenoids have several functions, ranging from supporting light harvesting during photosynthesis, to protecting the cell from high light exposure, and the structural support of the photosynthetic apparatus [6]. They are important for the cells to survive UV radiation, oxidative stress, and water or salt stress. These properties make carotenoids interesting for industrial applications, for example as food colorants, nutritional supplements, cosmetics, or health purposes. Especially in fungi, oxidative stress protection seems to be the major function and is intensively investigated [6]. Furthermore, fungi and yeasts were recently screened for commercial carotenoid production. Several Basidiomycetous yeast genera (e.g., *Rhodotorula* or *Phaffia*) were found to be candidates for a commercial utilization [7,8,9]. Interestingly it seems that extremophilic fungi produce higher amounts and a higher diversity of carotenoids [10,11,12,13]. One important group of extremophilic fungi are the so called black fungi or black yeasts, which can cause human diseases. Such yeasts can be found in almost any environment and often form subaerial biofilms (SABs) [14,15,16,17]. Among black fungi, several salt and cold tolerant fungi were found, such species could be candidates for industrial carotenoid production [10,12]. The most obvious feature of black fungi is their dark pigmentation, which results from inclusion of melanin into the cell wall. Formation of melanin together with the production of secondary metabolites including carotenoids and mycosporines, are passive physiological adaptations that help these fungi resisting environmental stresses [18,19,20,21,22]. Secondly black fungi are characterized by a thick cell wall providing a high mechanical protection especially under water stress [22].

The rock inhabiting fungus *K. petricola* A95(syn. *Sarcinomyces petricola*), which was isolated from a marble rock near the Philopappos monument on Musaios Hill, Athens (Greece) [23] belongs to an ancestral lineage of the order Chaetothyriales [24] and possesses all characteristic features of black fungi, including meristematic growth, melaninized cell walls and extensive secondary metabolite production [22]. The desiccation resistant black yeast *K. petricola* A95 is recently under investigation regarding stress response mechanisms and adaptation processes [25]. Since it produces melanin, carotenoids and mycosporins [21], among other stress related substances, it is an ideal candidate to investigate the role of the single components regarding stress response in black fungi. The high variety of carotenoids in fungi and the growing fungal related biotechnology demand techniques and protocols to investigate pigment composition and production under various environmental conditions. Recent reviews show the growing interest of biotechnology industry to produce such pigments in yeast cultures or other fungi and listed important factors (carbon source, aeration, light etc.) and limiting steps (production rates, enzymatic activity etc.) in carotenoid production [7,8,26]. To analyze carotenoid concentration, accumulation, and conversion a highly efficient, cost effective reliable system with a low detection limit is needed. HPLC is one of the standard methods used for such analyses. However, sample preparation, especially the difficulties of extracting specific carotenoids (low concentration or derivatives), requires a specific sample treatment (cell disruption), extraction medium, and solvent mixture. Several studies discussed separation of xanthophylls with HPLC, but they reported difficulties for some carotenoids. Most studies propose a C18 column in combination with a wide variety of solvents; e.g., studies used water-acetone mixtures [27,28], acetonitrile or hexane as extraction medium or solvent. All of the extraction solutions or solvents face the same problem: that they are not equally suitable for all pigments. Additionally, a comparison of carotenoids by spectroscopic measurements is difficult, since the absorption spectra depend on the solvent used [29] and the solvents can chemically react with pigments and such reactions can result in pigment derivatives [30].

Several methods were tested to increase extraction efficiency, mechanical or chemical treatment, and the use of specific solvents. The enhancement of pigment extraction was demonstrated by using mechanical or chemical treatment [31,32], ultra-sonication for cell disruption, and by using different extraction media [33]. Cell consumption is critical for quantitative pigment analysis. As [34,35] demonstrated the extraction efficiency depends on two parameters: an efficient cell disruption and a proper extraction medium. Mechanical treatment seems to be the preferred method for cells with thick cell walls [31,32]. Other parameters that influence pigment stability during extraction and therefore should be considered are pH-values, oxygen concentration, light and temperature.

The main fungal pigments are β-carotene, astaxanthin, lycopene, and neurosporene. Recently the production of torulene and torularhodin in fungi has gained some interest, especially as stress response to UV radiation [10,36,37]. The exact function of carotenoids in fungi is still under discussion, since mutants lacking carotenoids display the same growth rates as wild type strains. In the cases investigated, a lack of carotenoids has no apparent phenotypic consequences on growth or morphology in laboratory cultures. However, secondary effects of carotenoids, especially in regard to membrane integrity, are under discussion [6]. It is known in fungi that carotenoid formation is activated by blue light and H_2_O_2_, which generates oxidative stress [38]. The rock inhabiting fungus *K. petricola* A95 produces: β-carotene, γ-carotene, phytoene, torulene, and torularhodin and desiccation/rehydration stresses affect the formation of the colorless phytone as well as the other carotenoids [22]. A new pigment extraction protocol was used to investigate the carotenoid composition of the rock inhabiting yeast *K. petricola* A95 under control conditions but also under temperature and oxidative stress. This new, robust, and reproducible method allows quantitative analysis of carotenoids in black yeasts to be used to determine the adaptation responses to extreme environmental conditions in high stress resistant fungi.

## 2. Material and Methods

Cell cultures of *K. petricola* A95 were grown on MEA (malt extract agar) plates at 25 °C and 4 °C. Cell colonies were harvested after 7 days and freeze dried. Oxidative stress related pigment accumulation was tested with cell cultures grown (equal cell number) in liquid BG11 (cyanobacteria media,) media with 2% malt extract and 0.2% glucose and with subsequent addition of 20 mM H_2_O_2_ for 2 h. *K. petricola* A95 was cultivated in BG11 media to investigate biofilm formation and interaction with cyanobacteria as described in [39], especially since such biofilms are exposed to harsh environmental conditions, leading to adaptations in pigment compositions in both organisms.

All cells were freeze dried after harvesting (Labconco FreeZone 2.5), dry weights of the cells were determined and the cells were homogenized. A mill for glass beads with integrated CO_2_ cooling was used for the extraction of the pigments. The freeze dried material was transferred into a glass vessel with ground glass joint (Sartorius). The glass beads used had a mixing ratio of 1:3 (*w*/*w*), including 1 part of cells with a diameter of 1 mm and 3 parts of beads with an average diameter of 0.25 mm (Carl ROTH GmbH). Simultaneously the samples were disrupted with 100% HPLC grade acetonitrile (Sigma-Aldrich) for 3 min. To reduce the amount of fine particles in the supernatant, the samples were centrifuged at 20,800 g (Centrifuge 5417 C, Eppendorf) twice for 1 min after the disrupting procedure. The remaining pellets were tested for extraction efficiency by applying extraction solvents afterwards, to make sure, the pigment extraction was complete.

The determination of the different carotenoids was realized via HPLC (DIONEX, Ultimate 3000) A reversed phase C_18_ column (300-5, C18-250 × 4 mm, from CS Chromatography Service) was used. A two eluent protocol was used with following gradient (Table 1)

Eluent A was composed of 80% acetonitrile (HPLC grade; Sigma-Aldrich) and 20% aqua dest. (*v*/*v*) and Eluent B was 100% ethyl acetate (HPLC grade; Sigma-Aldrich). The total protocol is 30 min long and was carried out with a flow rate of 0.8 mL/min. The used solvents were degassed for 15 min by ultrasonication prior use.

Pigment identification and quantification was established for lycopene, didehydrolycopene, dehydrolycopene, β-carotene, γ-carotene, torulene, and as well for torularhodin. Authentic standards were obtained for β-carotene and lycopene from Sigma-Aldrich. The quantification was based on the β-carotene calibration curve, only, because lycopene is not soluble in the extraction solvent. Lycopene was dissolved in 100% hexane (HPLC grade; Sigma-Aldrich). Calculation of pigment concentrations were based on β-carotene calibration and dry weight as reference values. Therefore, peak areas were related to β-carotene concentration and then divided by the specific extinction coefficients of the pigments in acetonitrile [40,41]. The pigments were detected at 440 nm for β-carotene, at 460 nm for of γ-carotene, lycopene and dehydrolycopene at 470 nm, torulene at 480 nm and torularhodin was detected at 497 nm. The dry weight was subsequently included. During the HPLC measurements, absorption spectra were ascertained by the HPLC-detector itself, supported by a tungsten lamp. The absorption spectra were determined from 402 to 762 nm. Furthermore, concentrations of purchased standards (β-carotene and lycopene) were verified by photometric measurements (Carl Zeiss Specord M500), to prevent overloading of the column. In all determined conditions three different biological replicates with a dry weight of about 0.1 g were analyzed. Pigment concentrations were subjected to the one-way analysis of variance (ANOVA) test in order to establish comparisons between different stress treatments. All datas generated or analyzed during this study are included in this published article.

## 3. Results

**Figure 1:**
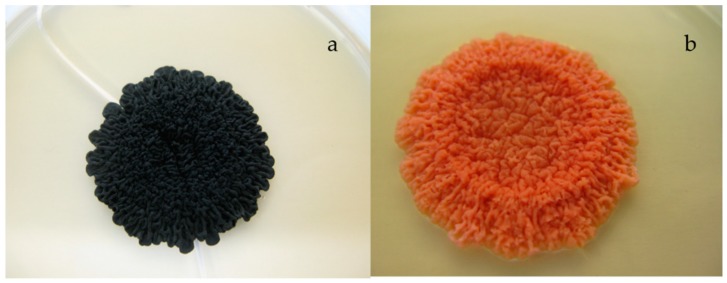
Colonies of *K. petricola* A95 (**a**) WT and (**b**) the spontaneous melanin deficient mutant (mdK) grown on MEA plates for 14 days at 25 °C. Colony size approximately 0.5 cm.

Pigment extraction was carried out with *K. petricola* A95 WT in comparison to a spontaneous melanin deficient mutant of *K. petricola* A95 (hereafter mdK) (Figure 1).

The mutant allowed us to determine the quality of the carotenoid extraction since the containing carotenoids were visible in this mutant and not masked by melanin as in the WT of *K. petricola* A95. Pigment extraction was carried out for freeze dried cell pellets with a minimum weight of 15 mg. Torularhodin was the pigment with the lowest concentration and minimum weight of the used material was adjusted to determine the specific amount of torularhodin accordingly. The thick cell wall of *K. petricola* A95 caused the use of relative long cell disruption times, and the shorter extraction lead to a higher variation in extraction efficiency. Therefore to ensure quantitative pigment extraction, cell pellets were tested for the remaining pigments in a second extraction step with 100% acetonitrile and 100% hexane. No significant amounts of pigments were detectable via HPLC analysis (<2 µg/g dry weight), once the method was established. The only pigment left in the pellet was melanin in the WT. The efficient pigment extraction after cell disruption was confirmed by using mdK of *K. petricola* A95, leaving a pale pellet after extraction. Ideally, pigment extraction and cell disruption must be carried out under low temperatures and in the dark without oxygen to prevent pigment degradation.

To verify the usability of our system the described standards of all the pigments were tested in our system. We determined the retention times of β-carotene and lycopene. Although lycopene was not completely soluble in 100% acetonitrile, a mixture of 105:30:25 (*v*/*v*/*v*) hexane, acetone and acetonitrile was used for this standard. A second, isocratic 100% hexane, gradient was established and in some cases the isocratic separation with hexane was used for the re-extraction of the pellets.

Several extraction solvents as well as HPLC separation set-ups were tested. Chemical extraction, for 2 h at 60 °C with either dimethyl sulfoxide or *n*-propanol, was proven to be not successful. Although a high extraction efficiency for both solvents was expected according to [34] and [42] and DMSO was frequently used for studies with fungi [43]. In this study, acetone was used with the described mill with integrated CO_2_ cooling combined with the Precellys 24 of Peqlab (at 6000 rpm for 5 up to 10 min) to ensure total cell disruption. In the latter cases the heating of the samples due to the rotation was the biggest problem and since sample cooling is crucial for stable pigment analysis, the mill was used exclusively. Furthermore, a solvent mixture of 40:20:40 (*v*/*v*/*v*) of acetonitrile, ethyl acetate, and *n*-propanol was tested, this mixture yielded good extraction results, but interacted negatively with the HPLC separation.

HPLC separation was tested with three eluents, acetonitrile/water, ethyl acetate, and ethyl acetate/acetone. Due to problems with a drifting baseline, eluents were reduced to eluent A (acetonitrile/distilled water) and eluent B (100% ethyl acetate). Eluent A was adapted, consisting of 80% acetonitrile (HPLC grade; Sigma-Aldrich)/20% distilled water. (*v*/*v*) was used. In combination with 100% acetonitrile as the extraction solvent the developed protocol ensures the detection of all pigments. All pigments show separate and distinct peaks. This was the most effective in extracting of the carotenoids from *K. petricola* A95 and was used as the established method. The total carotenoids in this approach were the highest, changing from, for example 153.7 µg g cdw^−1^ to 461.1 µg g cdw^−1^.

**Figure 2:**
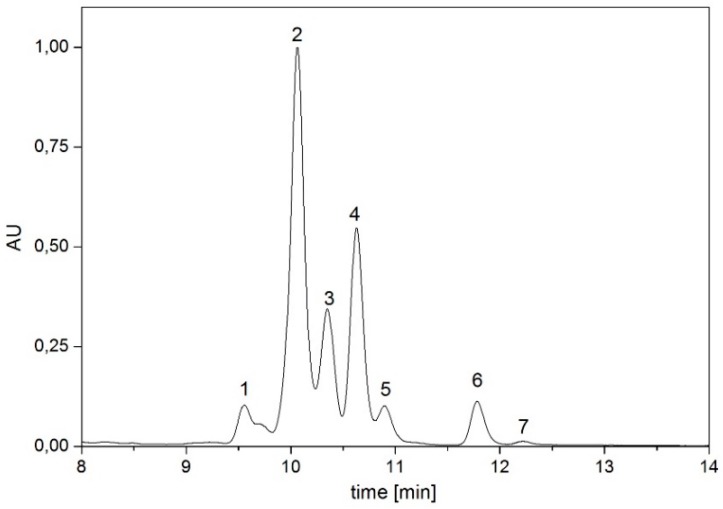
Representative HPLC chromatogram of carotenoids in *K. petricola* A95 detected in our experiments. The peaks were determined via reference substances and identified as dehyrolycopene (1), didehydrolycopene (2), torulene (3), lycopene (4), γ-carotene (5) and β-carotene (6) and torularhodin (7).

**Figure 3:**
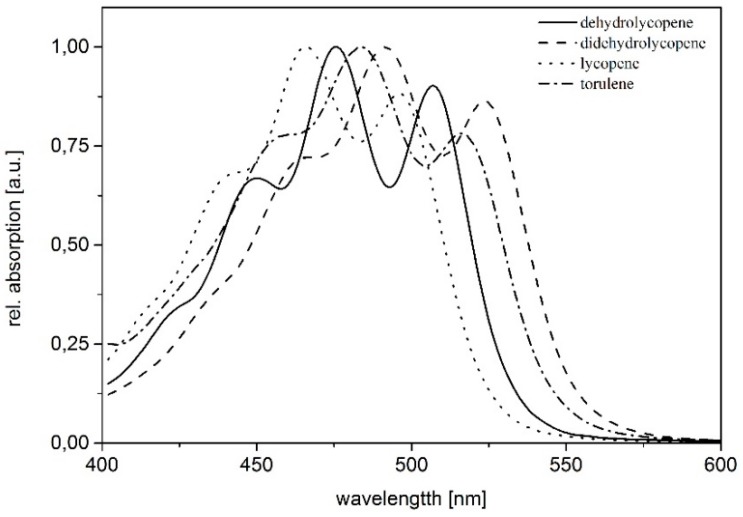
Absorption spectra of main carotenoids in *K. petricola* A95 (determined via HPLC detector in according eluent mixture). The maxima of each pigment was determined: didehydrolycopene (463 nm, 492 nm, 523 nm); torulene (454 nm, 484 nm, 515 nm); dehydrolycopene (448 nm, 476 nm, 507 nm); lycopene (440 nm, 465 nm, 496 nm).

In the final analysis, seven different pigments could be observed and quantified (Figure 2) in *K. petricola* A95. The pigments are determined in the following order: dehyrolycopene (1), didehydrolycopene (2), which was always the largest peak, torulene (3), lycopene (4), γ-carotene (5) and β-carotene (6). The pigment torularhodin (7) was only detectable after prolonged incubation under low temperatures in trace amounts. Standards and absorption spectra were used to confirm the identity of the different pigments (Figure 2 and Figure 3 and Table 2).

**Figure 4:**
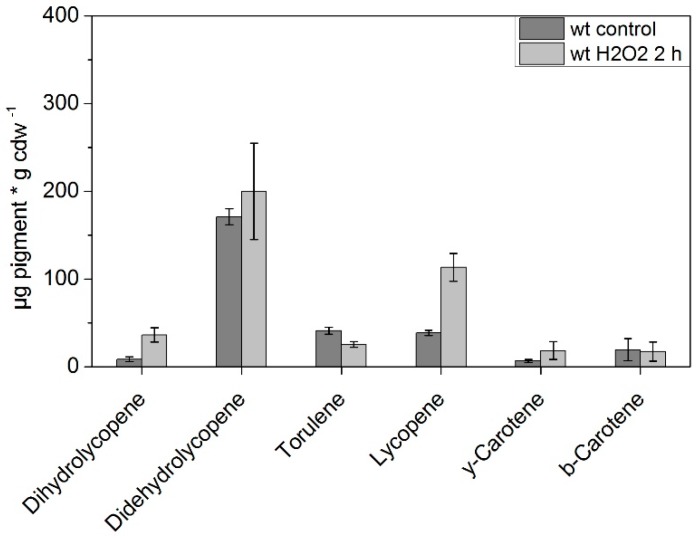
Carotenoid composition of WT *K. petricola* A95 incubated with and without 20 mM H_2_O_2_ for 2 h, determined via HPLC analysis (*n* = 3). Significant differences at *p* < 0.05 with respect to the control are marked by asterisks (one-way ANOVA).

Short-term incubation (2 h) of WT *K. petricola* A95 under oxidative stress (treatment with 20 mM H_2_O_2_) showed an increase in concentration for several carotenoids compared to growth under normal conditions for the same time period (Figure 4). The concentrations of lycopene (38.7 to 113.2 µg g cdw^−1^), γ-carotene (6.6 to 18.2 µg g cdw^−1^) and dihydrolycopene (8.5 to 36.2 µg g cdw^−1^) increased strongly under the applied conditions (differences significant for *p* = 0.05). The concentrations of β-carotene and torulene did not increase during the incubation and for didehydrolycopene an increase was detectable, however, the high variation between the samples did not allow us to draw conclusions. Didehydrolycopene showed in general the highest variation in pigment concentration in all samples.

**Figure 5:**
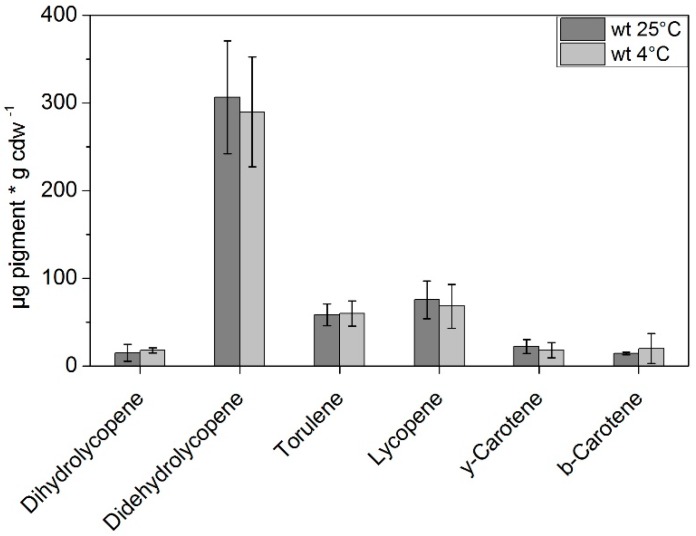
Pigment composition of *K. petricola* A95 (WT) determined via HPLC after growth under normal conditions (25 °C) and under low temperature (4 °C) for 7 days (*n* = 3). Significant differences at *p* < 0.05 with respect to the wild type are marked by asterisks (one-way ANOVA).

Incubation under low temperature did not influence the pigment composition (Figure 5), compared to cultures grown under normal temperatures (the ANOVA Test showed no significant differences between the control samples and temperature stressed cells for all pigments). However, a strong increase in the didehydrolycopene concentration (up to 300 µg g cdw^−1^) under both conditions was visible after long term incubation. Therefore, the total measured pigment concentration increased to ~500 µg g cdw^−1^. Identical results for all experiments are exhibited for the spontaneous mdK of *K. petricola* A95. The spontaneous mutant displays a similar amount of pigments per dry weight and the carotenoid concentrations did not change as a response to temperature stress (data not shown).

## 4. Discussion

For an efficient pigment analysis, two steps are important: First, a solvent for extraction should be selected that allows the quantitative determination of all relevant pigments. In specific cases and with unknown samples, a selection and/or a mixture of media should be used, like acetonitrile, ethyl acetate, isopropanol, and hexane. The most suitable solvents seemed to be isopropanol and hexane, which were used to verify the extraction efficiency (re-extraction of the pellet), but caused a baseline shift in the HPLC. It is noteworthy to state that DMSO is to our opinion not a suitable extraction solvent; the difficulties to remove DMSO from the system limit the application in an adequate eluent system. After centrifugation and removal, the supernatant residues of DMSO still remain in the sample, which is problematic for HPLC analyses. Additionally, the proposed DMSO based method of pigment extraction [37,44] should be tested accordingly, depending on the systems used, to ensure optimal pigment analysis results. Thus, DMSO extraction is always a time consuming method and the results are hardly comparable with other methods. Secondly an efficient cell rupture method under conditions that ensure the pigment stability should be selected. Black yeasts are characterized by a thick cell wall with high melanin concentration. In general pigment extraction should be performed under low oxygen, and if possible under oxygen free conditions. The major challenge in pigment analysis is to recover pigments with minimum risk of damage but with high efficiency. A mechanical treatment is needed in the most cases but the method applied should be tested carefully due to the high sensitivity of the molecules. Especially for black yeasts and other fungi with a thick cell wall, a mechanical cell disruption seems necessary and provides better results as a chemical treatment [31,32].

*K. petricola* A95 displayed an uncommon pigment composition; especially in the high concentrations of dihydrolycopene, didehydrolycopene and torulene, which are not so often described in fungi. A normally high abundance of carotene derivatives and lycopene can be observed in pigmented fungi [45,46]. Several yeasts showed a high torulene concentration but no dihydrolycopene was detected in yeast so far [46,47]. The abundance of didehydrolycopene and torulene indicates a similar production pathway as described for *N. crassa*, leading in this fungus to neurosporaxanthin [2,48], a carotenoid that was not detectable in *K. petricola* A95 under the applied conditions. It is noteworthy to mention that the determination of the abundance of astaxanthin and neurosporaxanthin is technically possible with the described protocol. The only pigment left in the pellet was melanin in the WT. Hence comparing the results with methods developed for pigments in algae or higher plants [26,27,31,49,50,51] is not advisable due to the thick cultured cell wall of the supervised fungi. Although [49] compared different methods using maize seeds, they are not characterized by a thick cell wall like black yeast. Similar to our study, the decision regarding efficacy was made with respect to the color of the pellet. In general, these studies provide a wide method spectra and were useful regarding extraction, elution, and calibration of the authentic standards, see also [31,51]. Fungi pigments were mainly analyzed, focusing on certain valuable pigments only. Knowledge regarding pigment composition in fungi is needed for species description, however, such studies used standard methods and did not compare different methods [5,6,7,10,33,34,52]. Methodological overviews for fungi pigment extraction are still missing [38]. Therefore, the established method was compared to [19]. The combination of extraction media and the eluent system of [19] resulted in a strong injection peak in the presented HPLC system and was adjusted afterwards, concerning the different tested method combinations shown.

The detected carotenoids are consistent with [22], they found also carotene derivatives, torulene, and torularhodin. Our extraction method, especially the extraction medium and the mechanical treatment, gave us additionally the possibility to quantify lycopene and didehydrolycopene, unusual pigments in fungi and not often found in yeast strains.

To our opinion didehydrolycopene functions as a synthesis interface, comparable to β-carotene in photosynthetic organisms. Such a pool of didehydrolycopene can be converted fast to stress response pigments and helps cells to respond quickly to environmental stresses. The two main pigments in *K. petricola* A95, lycopene and didehydrolycopene, display much higher concentrations, as in other fungi detected [53] and β-carotene concentrations are lower compared to other fungi and yeasts. The detected lycopene derivatives are the major carotenoids in *K. petricola* A95. Future experiments must be carried out to find out if these pigments have a photoprotective role or if they function as a precursor for other pigments. In *N. crassa* the conversion of didehydrolycopene to torulene by cyclisation was described [54]. The already described torularhodin and torulene could be involved in photoprotection [6,13,37,55]. The antioxidant properties of torulene are attributed to its conjugated double bond system; in fact, torulene has more antioxidant efficiency than β-carotene, which presents less of a double bond on its chemical structure than torulene [56]. However, several other carotenoids were also described to have an antioxidant activity.

Another explanation for the stable carotenoid concentrations in *K. petricola* A95 would be that the mechanism of carotenoid action is more likely to consist of shielding sensitive molecules or organelles than of the neutralization of harmful oxidants [52]. Therefore, carotenoids do not play a major physiological role in fungal cells, but they may have beneficial effects under specific conditions. Several studies showed an increase in carotenoid concentration under stress [45,57]. The potential photoprotective pigment torulene [9,58] showed low concentrations in *K. petricola* A95, which would imply a less important role in *K. petricola* A95, but an inducible production under a stress situation out of the more stable dehydrolycopene seems possible. However, we could not detect an increase in torulene concentrations if the cells were exposed to oxidative or temperature stress. Such an induction was described for a black yeast under oxidative stress [59], in red yeast under salt stress [58], and a light dependent increase of the overall carotenoid concentration was described for *N. crassa* [38] incubated with H_2_O_2_. Such an inducible protective system would enable *K. petricola* A95 to react fast to stress situations. A similar system was described for the red yeast *Dioszegia* with the xanthophyll plectaniaxanthin [60]. The photoprotective carotenoid torularhodin was induced in *K. petricola* A95 under cold temperatures, but very low amounts and other stresses or longer incubation times have to be investigated to unravel the function of this pigment in *K. petricola* A95.

The major changes under the applied conditions were detected for lycopene and derivatives, which should therefore be considered as the most important pigments in *K. petricola* A95. The high concentration of the unusual carotenoid didehydrolycopene in *K. petricola* A95 should be investigated in detail, especially since the photoprotective role and the biotechnological potential of lycopene derivatives was described before [49]. Screening other black yeasts regarding their pigmentation could result in a wide diversity of pigments with multiple promising applications. Black fungi are remarkable in their stress resistance and we showed that their carotenoid pigmentation is complex and future studies have to be performed to determine the specific function of the specific pigments. The protocol presented will allow the quantitative analysis of black fungi, characterized by thick cell walls and melanin pigmentation, regarding stress response and adaptation to extreme environmental conditions. Future experiments have to unravel the function of melanin, carotenoids, and the cell wall structure regarding the stress responses in detail.

## 5. Conclusions

Extremotolerant and extremophile black yeasts are a promising source of pigments and other chemicals. New protocols and studies are needed to determine the capacity for production of these high stress resistance fungi. The established protocol allows the determination of carotenoid composition in black yeasts. Oxidative stress results in an adaptation in pigment composition. Future experiments have to be carried out to determine if didehydrolycopene functions as a protective agent itself or if it serves as a precursor for antioxidative pigments like torulene and torularhodin, which could be produced after induction under stress conditions.

## Figures and Tables

**Table 1:** Eluent protocol used for carotenoid separation.

Time [min]	Eluent A	Eluent B
0	60%	40%
7	50%	50%
17	40%	60%
21	30%	70%
28.5	20%	80%
29.5	10%	90%
30.5 to 42 min	60%	40%

**Table 2:** Pigments absorption maxima and concentrations of the black yeast *Knufia petricola* A95 grown on MEA plates under standard growth conditions.

Pigment	Absorption Maxima [nm]	Concentration [µg/g dry weight]
dihydrolycopene	448, 476, 507	30.74
didehydrolycopene	463, 492, 523	383.12
torulene	454, 484, 515	85.39
lycopene	440, 465, 496	100.30
γ-carotene	460, 489	23.88
β-carotene	457, 483	7.53
torularhodin	469, 497, 525	5.84
